# Development of an interpretable machine learning model for Ki-67 prediction in breast cancer using intratumoral and peritumoral ultrasound radiomics features

**DOI:** 10.3389/fonc.2023.1290313

**Published:** 2023-11-17

**Authors:** Jing Wang, Weiwei Gao, Min Lu, Xiaohua Yao, Debin Yang

**Affiliations:** Departments of Ultrasound, Jiading District Central Hospital Affiliated Shanghai University of Medicine & Health Sciences, Shanghai, China

**Keywords:** breast cancer, Ki-67 levels, radiomics, peritumoral ultrasound segmentation, machine learning, support vector machine

## Abstract

**Background:**

Traditional immunohistochemistry assessment of Ki-67 in breast cancer (BC) via core needle biopsy is invasive, inaccurate, and nonrepeatable. While machine learning (ML) provides a promising alternative, its effectiveness depends on extensive data. Although the current mainstream MRI-centered radiomics offers sufficient data, its unsuitability for repeated examinations, along with limited accessibility and an intratumoral focus, constrain the application of predictive models in evaluating Ki-67 levels.

**Objective:**

This study aims to explore ultrasound (US) image-based radiomics, incorporating both intra- and peritumoral features, to develop an interpretable ML model for predicting Ki-67 expression in BC patients.

**Methods:**

A retrospective analysis was conducted on 263 BC patients, divided into training and external validation cohorts. From intratumoral and peritumoral regions of interest (ROIs) in US images, 849 distinctive radiomics features per ROI were derived. These features underwent systematic selection to analyze Ki-67 expression relationships. Four ML models-logistic regression, random forests, support vector machine (SVM), and extreme gradient boosting-were formulated and internally validated to identify the optimal predictive model. External validation was executed to ascertain the robustness of the optimal model, followed by employing Shapley Additive Explanations (SHAP) to reveal the significant features of the model.

**Results:**

Among 231 selected BC patients, 67.5% exhibited high Ki-67 expression, with consistency observed across both training and validation cohorts as well as other clinical characteristics. Of the 1698 radiomics features identified, 15 were significantly correlated with Ki-67 expression. The SVM model, utilizing combined ROI, demonstrated the highest accuracy [area under the receiver operating characteristic curve (AUROC): 0.88], making it the most suitable for predicting Ki-67 expression. External validation sustained an AUROC of 0.82, affirming the model’s robustness above a 40% threshold. SHAP analysis identified five influential features from intra- and peritumoral ROIs, offering insight into individual prediction.

**Conclusion:**

This study emphasized the potential of SVM model using radiomics features from both intra- and peritumoral US images, for predicting elevated Ki-67 levels in BC patients. The model exhibited strong performance in validations, indicating its promise as a noninvasive tool to enable personalized decision-making in BC care.

## Introduction

The Ki-67 antigen is a well-established marker in cell proliferation, essential in categorizing luminal subtypes of tumors and predicting therapeutic outcomes in breast cancer (BC) (1, 2). Higher expression levels signify increased aggressiveness, risk of recurrence, and poor prognosis (3). The traditional approach to preoperative assessment of Ki-67 involves immunohistochemistry, requiring tissue samples usually extracted by core needle biopsy (CNB), and subsequent visual analysis by a pathologist (4). However, this primary method is invasive, time-consuming, and nonrepeatable. The inherent heterogeneity of BC results in a concordance rate between CNB and excision specimen, with a substantial variation ranging from 59-88% (5, 6). Additionally, the inability of the traditional approach to dynamically evaluate Ki-67 changes during neoadjuvant therapy highlights its limitations (7). Therefore, a method that is noninvasive and capable of continuous monitoring is urgently needed for the clinical evaluation of Ki-67 status.

With the accelerated advancement of artificial intelligence (AI) techniques, machine learning (ML) has marked remarkable progress in image processing and feature mining, particularly in classification tasks for benchmark images (8). This technology has been transformative; however, its efficacy relies heavily on large sample sizes, making it challenging in medical applications where extensive data extraction from individual patient images is needed (9). Addressing this challenge, radiomics emerged as a solution. This field involves the high-throughput extraction and analysis of vast quantities of quantitative features from digital images, transcending the limitations of human visual perception (10, 11). By identifying correlations between these imaging features and underlying tissue information, radiomics can enhance performance in evaluating the biological characteristics and prognosis of tumors, thus contributing to the optimization of complex clinical decision-making processes (12).

Building upon the significant advancements in the field of radiomics, research has predominantly centered on utilizing magnetic resonance imaging (MRI) to predict Ki-67 levels within BC tissues (13–15). In contrast, ultrasound (US)-based radiomics for the prognostication of Ki-67 remains relatively unexplored. US imaging offers notable advantages over MRI, including wider accessibility, cost-effectiveness, suitability for repeated examinations, superior spatial resolution, real-time availability, and the absence of contraindications for specific patient conditions. In the last few years, a limited number of studies have explored the application of US radiomics in predicting Ki-67 levels. While these investigations represent an encouraging development, they have typically demonstrated a modest predictive efficacy, with the area under the receiver operating characteristic curve (AUROC) usually ranging between 0.7 to 0.8 (16–18). The underlying reason may be the prevalent focus on intratumoral features, neglecting critical biological insights available in the peritumoral area and thus potentially constraining the predictive accuracy of radiomics models. Interactions within the peritumoral area can influence tumor evolution and progression (19), such as inducing cytokine release that fosters an immunosuppressive microenvironment (20). Additionally, peritumoral factors like edema and angiogenesis have been correlated with tumor malignancy (21, 22), indicating that integrating intra- and peritumoral regions in radiomics analysis may enhance predictive capabilities.

In light of these considerations, the present study aims to investigate the potential of US-based radiomics, utilizing both intratumoral and peritumoral regions, in establishing an interpretable ML model for predicting Ki-67 expression in BC patients, thereby contributing to individualized treatment strategies and prognosis assessments.

## Materials and methods

This study was conducted in accordance with the ethical guidelines of the Declaration of Helsinki and received approval from the Institutional Review Board of Jiading District Central Hospital Affiliated Shanghai University of Medicine & Health Sciences (2023K29). Due to the retrospective nature, the requirement for informed consent was waived, and all patient data were carefully anonymized.

### Patient selection

The study incorporated a comprehensive review of medical records from January 2018 to July 2023, resulting in the identification of 263 female BC patients who met specific criteria. Inclusion in the study required candidates to satisfy the following: 1) a surgical resection-confirmed diagnosis of invasive ductal carcinoma; 2) the presence of a singular and mass-formed breast tumor; 3) Ki-67 status verification through both CNB and excision specimen; 4) US evaluations performed within two weeks before surgery. Additionally, exclusion criteria encompassed: 1) inadequate US imagery or incomplete lesion display; 2) a history of preoperative treatments such as radiotherapy, chemotherapy, or neoadjuvant therapy; 3) an absence of comprehensive clinical details. After rigorous screening, the selected BC patients were divided into training and external validation cohorts in a 7:3 ratio, ensuring the credibility of the predictive model. Clinical and histopathological data, including key aspects such as Ki-67 expression, tumor diameter, and breast imaging-reporting and data system (BI-RADS), were retrieved from medical records. The Ki-67 expression was quantified using the St. Gallen International Expert Consensus guidelines (23). A threshold was set, classifying samples with Ki-67 values of ≥14% as high expression level and those below this value as low expression level.

### Image acquisition and segmentation

**Figure 1:**
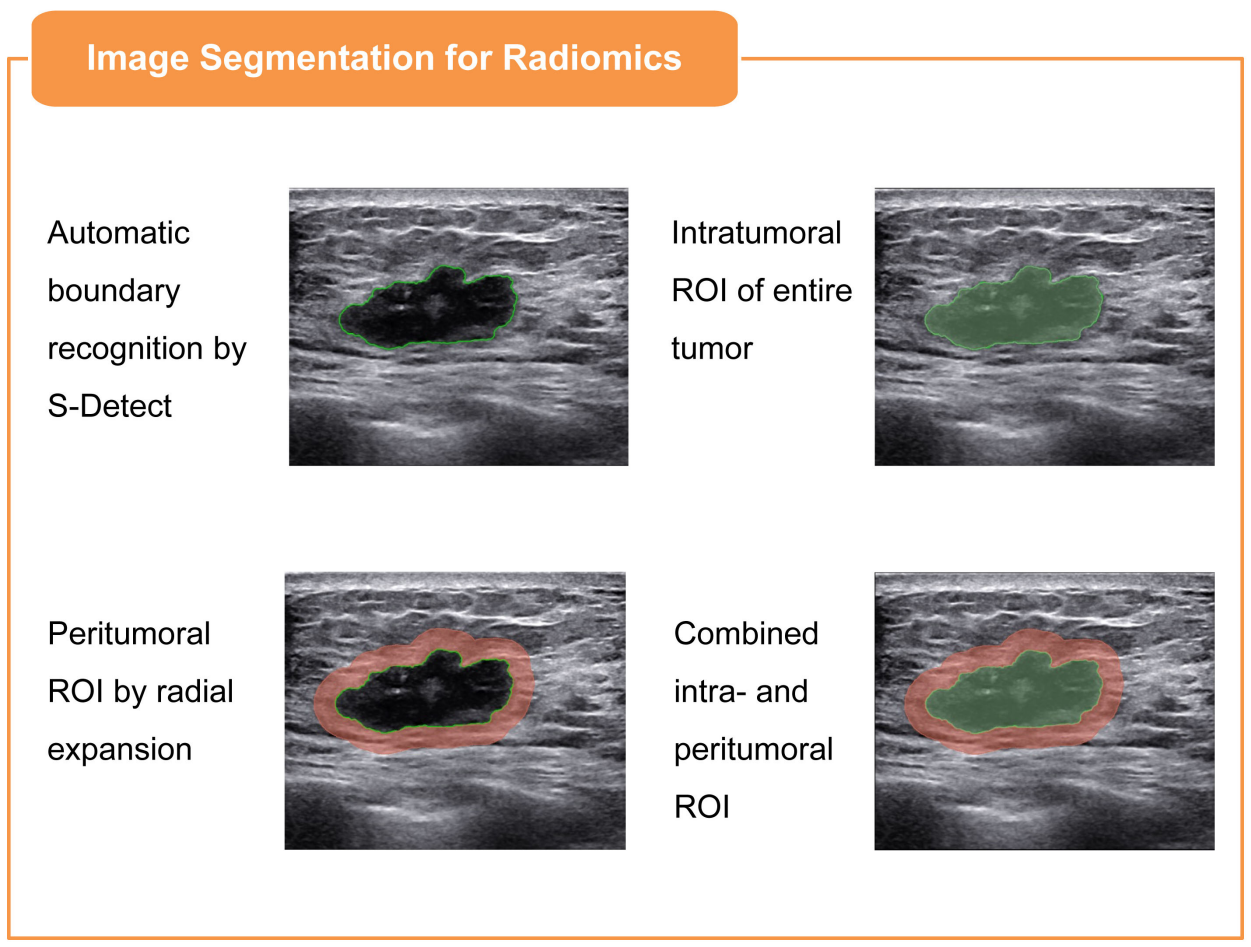
Illustration of three ROIs in BC ultrasound imaging: intratumoral, defined via S-Detect mode; peritumoral, derived from the intratumoral ROI by 3mm radial expansion and subtraction; and combined, an integration of both intratumoral and peritumoral ROIs.

Bilateral breast US examinations were conducted following the standard scanning protocol with a Samsung RS80A ultrasound system (Samsung Medison, Co. Ltd., South Korea), employing an L3-12A linear array probe. Both longitudinal and transverse sections were captured and saved in the Digital Imaging and Communications in Medicine (DICOM) format for subsequent evaluation. For the region of interest (ROI) segmentation in the radiomics analysis, two senior sonographers, each with over 15 years of expertise in BC ultrasonography, were assigned to use the system’s built-in S-Detect mammary gland mode to automatically recognize the tumor boundaries, ensuring segmentation reliability. The image displaying the lesion at its maximum diameter was selected as the input. Upon centering the lesion, the system autonomously delineated the lesion’s boundary, defining it as the ROI. If the automatically drawn border failed to align with the solid edge of the mass, the operator made manual adjustments to achieve the correct contour. After finalizing the most accurate boundary, the radiomics intratumoral ROI was segmented through the use of the open-source imaging platform 3D Slicer software (v5.0.2). In assessing the peritumoral areas, the intratumoral ROI was expanded radially by 3 mm from the tumor boundary to form a dilated ROI, with segments extending beyond the skin excised. This methodology was adopted in accordance with the insights from Ding et al. (24) regarding the optimal sizing of the peritumoral regions in radiomics analysis. The intratumoral ROI was then subtracted from the dilated ROI to derive the peritumoral ROI. Consequently, three distinct ROIs (intratumoral, peritumoral, and combined ROI) were identified for each patient, as illustrated in **Figure 1**.

### Radiomics feature extraction and selection

Subsequent to the precise segmentation of intra- and peritumoral ROIs, the radiomics features were systematically extracted utilizing the 3D Slicer radiomics extension. For both intra- and peritumoral ROIs, a total of 849 distinctive features were extracted for each modality. The original features were categorized into three main groups: 12 shape-based attributes, 18 first-order statistics, and 75 texture features. The texture features were further delineated into five specific matrices, comprising 24 gray-level co-occurrence matrix (GLCM) features, 14 gray-level dependence matrix (GLDM) features, 16 gray-level run length matrix (GLRLM) features, 16 gray-level size zone matrix (GLSZM) features, and 5 neighbouring grey tone difference matrix (NGTDM) features. Additionally, 744 filtered features were derived using wavelet transformations applied to the original first-order and texture attributes, enhancing the depth and complexity of the feature set.

Prior to feature selection, a crucial data preprocessing step was conducted to standardize features using Z-score normalization, aligning them to a mean of zero and a standard deviation of one. The predictive feature selection was carried out through a systematic three-step approach. Initially, interobserver reproducibility for each feature was evaluated by employing intraclass correlation coefficient (ICC) analysis, and a threshold of 0.85 was established for acceptable agreement, minimizing delineation discrepancies between the sonographers. Subsequently, the Student’s t-test was utilized to retain features manifesting false discovery rate-corrected P values below 0.05, identifying them as potential predictors. Lastly, the feature selection was further refined through the application of the least absolute shrinkage and selection operator (LASSO) logistic regression, focusing on the variables that were most representative of Ki-67 expression relationships.

### Development and internal verification of ML models

In the pursuit of predicting high Ki-67 expression, the study engaged in the creation of distinct ML models founded on intratumoral ROI, peritumoral ROI, and a synergistic combination of both. Utilizing logistic regression (Logit), random forests (RF), support vector machine (SVM), and extreme gradient boosting (XGBoost), the study applied a triply-repeated five-fold cross-validation strategy to each data subset during the model training phase. This method ensured a rigorous allocation of the data into designated training and testing segments, facilitating optimal model construction. Following the formulation of these models, an internal verification process was conducted to assess the models’ discrimination, calibration, and clinical applicability. The selection of the optimal predictive model was determined by its superior discriminative performance, robust calibration, and alignment with clinical utility.

### External verification and interpretability of the optimal model

The complete evaluation of the selected model commenced with external verification, focusing on the discriminative function, calibration, and applicability in an independent sample. This was followed by an interpretative analysis using the SHAP (shapley additive explanation) methodology to dissect the contributions of individual variables to the prediction (25). Stemming from cooperative game theory, SHAP facilitates the quantification of each feature’s individual impact on the model’s prediction by computing the average marginal contribution, thereby addressing the inherent ‘black box’ nature of ML models (26). By analyzing the significance of each feature and ranking them according to their respective SHAP values in descending order, the study identified key predictors, thereby enhancing the comprehension of the intricate relationships that influence Ki-67 expression within the examined patient cohort.

### Statistical analysis

A comprehensive statistical approach was adopted in line with the data characteristics. Comparisons between the training and external validation cohorts were made using chi-square tests, Mann-Whitney U tests, and independent-sample t-tests. Univariate and multivariate Logit analyses were used to identify clinical predictors associated with increased Ki-67 expression, and their joint predictive accuracy was assessed using the AUROC. In evaluating the ML model, the AUROC was used for discrimination, calibration curve analysis for model fit, and decision curve analysis (DCA) for net benefits. All statistical analyses were performed using IBM SPSS Statistics (v 22.0, SPSS Inc.) and Python (v 3.7.1).

## Result

### Patient information

**Figure 2:**
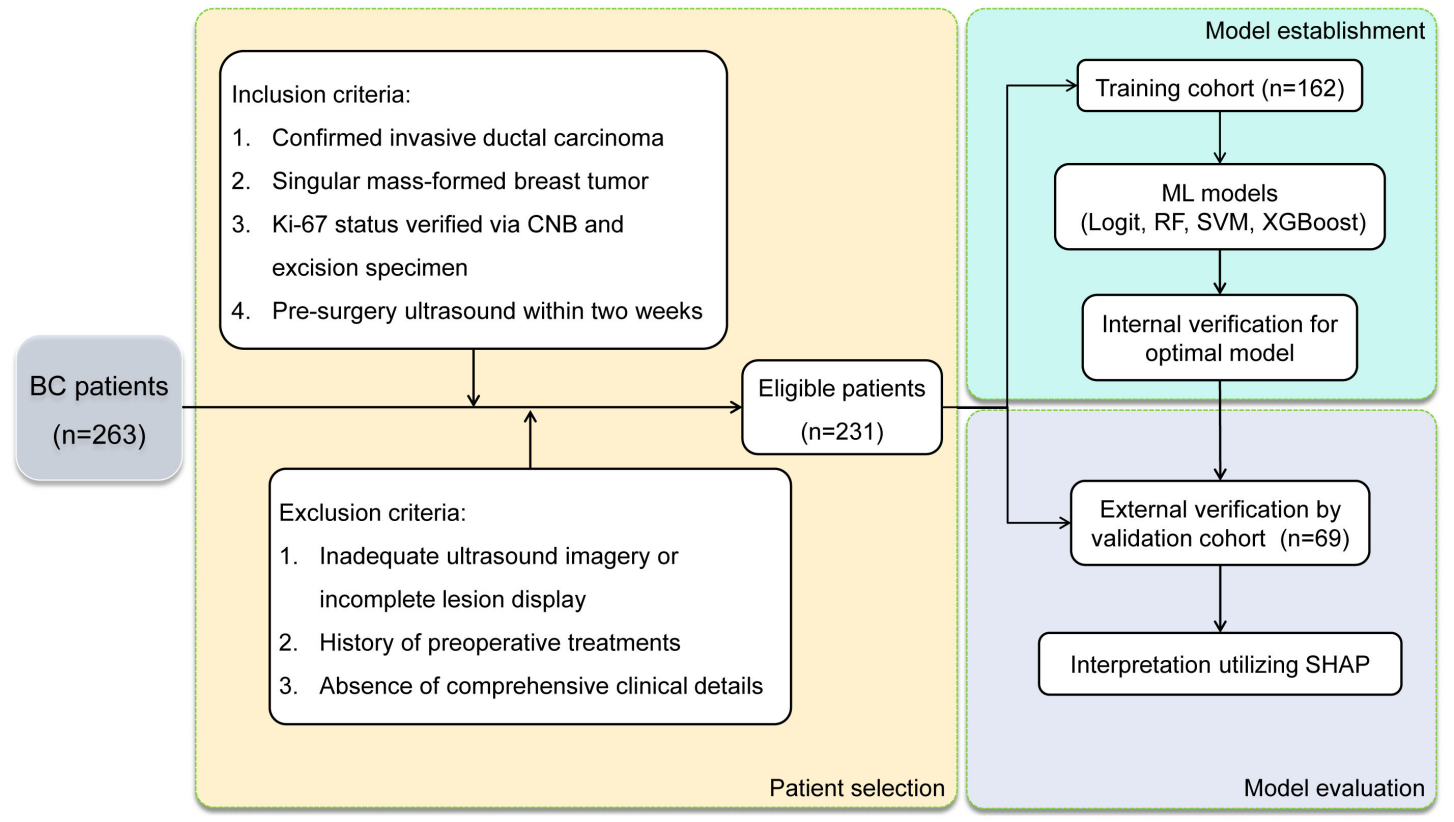
Flow diagram of patient selection process and model establishment & evaluation in BC patients.

The selection process yielded 231 BC patients meeting the inclusion and exclusion criteria, with high Ki-67 expression levels identified in 67.5% of the cases. These patients were divided into a training cohort (n=162) and an external validation cohort (n=69). High Ki-67 expression was identified in 67.9% and 66.6% of the patients, respectively, with no statistically significant difference (χ^2^ = 0.034, P = 0.854). **Figure 2** illustrates the selection process, model establishment, and model evaluation, while **Table 1** confirms an even distribution between cohorts without significant disparities in clinical characteristics (all P-values > 0.05).

**Table 1:** Clinical characteristics comparison between training and validation cohorts.

Characteristics	Training cohort (n=162)	Validation cohort (n=69)	P-value
Age (years)	56.26 ± 7.09	56.46 ± 7.37	0.846^a^
Menopausal status			0.878^b^
Premenopausal	67 (41.36%)	30 (43.48%)	
Postmenopausal	95 (58.64%)	39 (56.52%)	
Family history			0.956^b^
Absent	153 (94.44%)	66 (95.65%)	
Present	9 (5.56%)	3 (4.35%)	
Tumor diameter (mm)	20.60 ± 6.13	22.27 ± 5.35	0.050^a^
Location			0.116^b^
Left	95 (58.64%)	32 (46.38%)	
Right	67 (41.36%)	37 (53.62%)	
US-reported LN status			0.409^b^
Negative	132 (81.48%)	60 (86.96%)	
Positive	30 (18.52%)	9 (13.04%)	
High Ki-67 expression	110 (67.90%)	46 (66.67%)	0.976^b^
Molecular subtypes			0.850^b^
Luminal A	53 (32.72%)	24 (34.78%)	
Luminal B	62 (38.27%)	23 (33.33%)	
HER-2 positive	29 (17.90%)	15 (21.74%)	
Triple negative	18 (11.11%)	7 (10.14%)	
BI-RADS			0.628^c^
3	2 (1.23%)	1 (1.45%)	
4A	8 (4.94%)	3 (4.35%)	
4B	33 (20.37%)	15 (21.74%)	
4C	60 (37.04%)	28 (40.58%)	
5	59 (36.42%)	22 (31.88%)	

^a^for independent sample t-test, ^b^for chi-square test, and ^c^for Mann-Whitney U test.

### Identification of independent predictors for high Ki-67 expression

**Figure 3:**
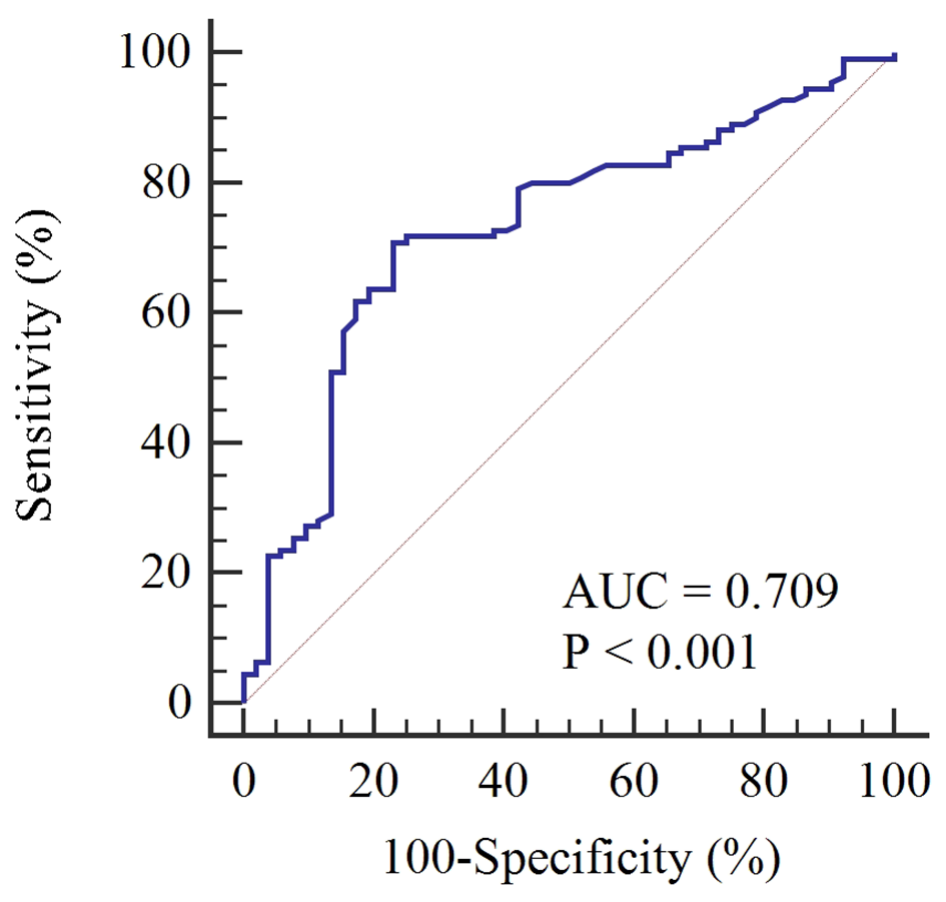
ROC analysis for the prediction of high Ki-67 expression based on age, tumor diameter, and US-reported positive LN, demonstrating moderate prediction accuracy with an AUC of 0.709.

**Table 2** describes the association between clinical characteristics and high Ki-67 expression through both univariate and multivariate Logit analyses. Age, tumor size, and US-reported positive lymph node (US-reported positive LN) were identified as the independent predictors (all P-values < 0.05). By applying these predictors in a Logit model, the ability to predict Ki-67 expression was found to be moderate, evidenced by an AUROC of 0.709 (**Figure 3**).

**Table 2:** Univariate and multivariate Logit analysis of clinical characteristics associated with high Ki-67 expression.

Characteristics	Univariate analysis			Multivariate analysis		
	P-value	OR	95% CI	P-value	OR	95% CI
Age	0.003	1.089	1.028-1.153	0.010	1.081	1.019-1.147
Menopausal status	0.863	0.942	0.482-1.844			
Family history	0.935	0.942	0.226-3.925			
Tumor diameter	0.005	1.088	1.025-1.154	0.020	1.076	1.012-1.145
Location	0.61	0.841	0.431-1.638			
US-reported positive LN	0.025	3.210	1.161-8.874	0.048	2.902	1.012-8.313
BI-RADS	0.959	1.009	0.71-1.435			

### Radiomics feature analysis

**Figure 4:**
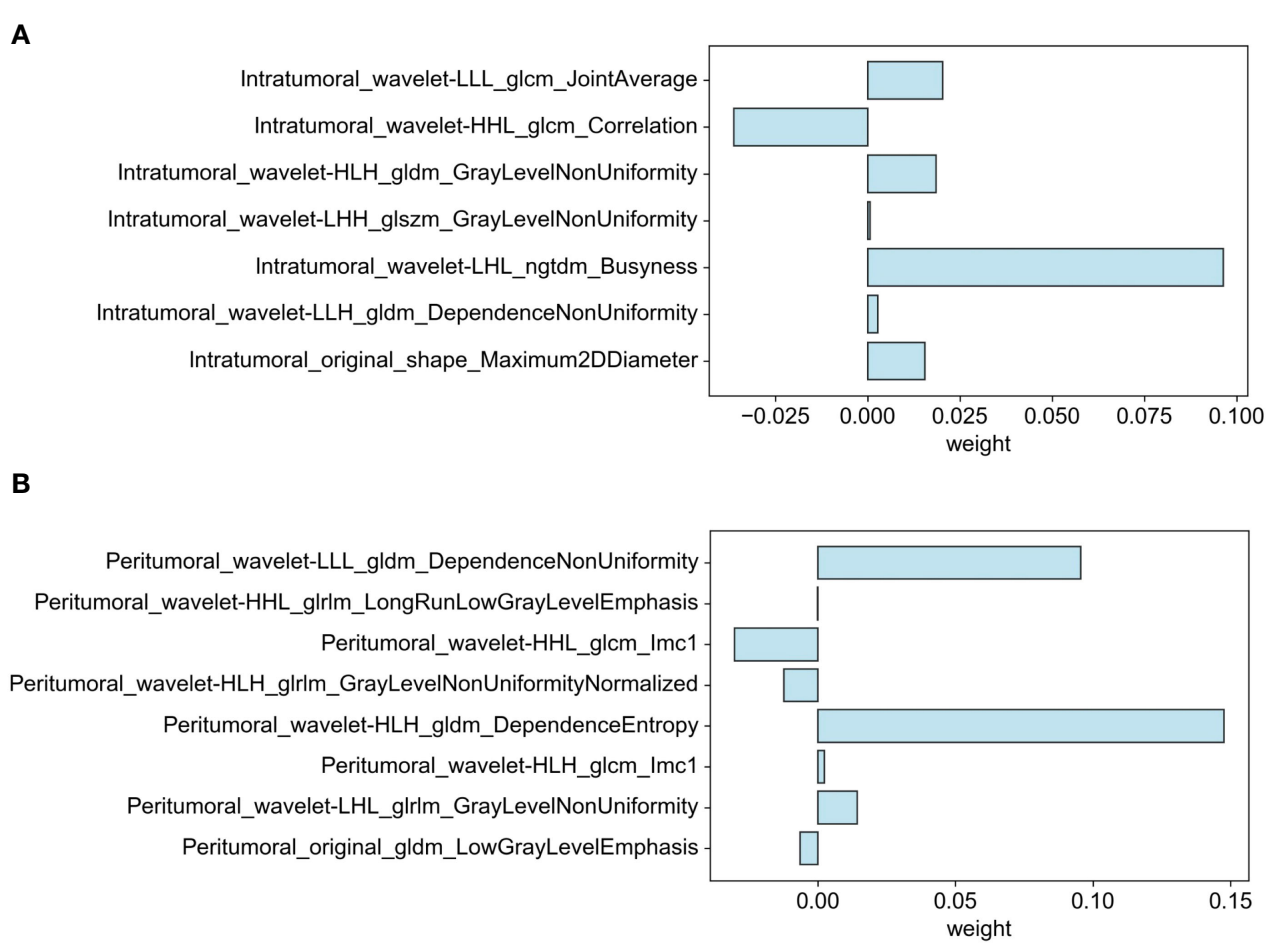
Distribution of selected features associated with Ki-67 expression: **(A)** Intratumoral segmentation and **(B)** Peritumoral segmentation using LASSO regression.

Through the process of segmenting intra- and peritumoral ROI in grayscale US from each patient in the training cohort, 1698 radiomics features were identified. Following normalization, 1158 features (68.2%) with intra-observer ICC of 0.85 or higher were retained for stability in subsequent analysis. The application of Student t-test identified 107 features potentially correlated with elevated Ki-67 levels. Final selection, utilizing LASSO regression, isolated 15 significant features associated with Ki-67 expression: 7 intratumoral and 8 peritumoral. These feature distributions are delineated in **Figure 4**.

### ML model establishment and selection

**Figure 5:**
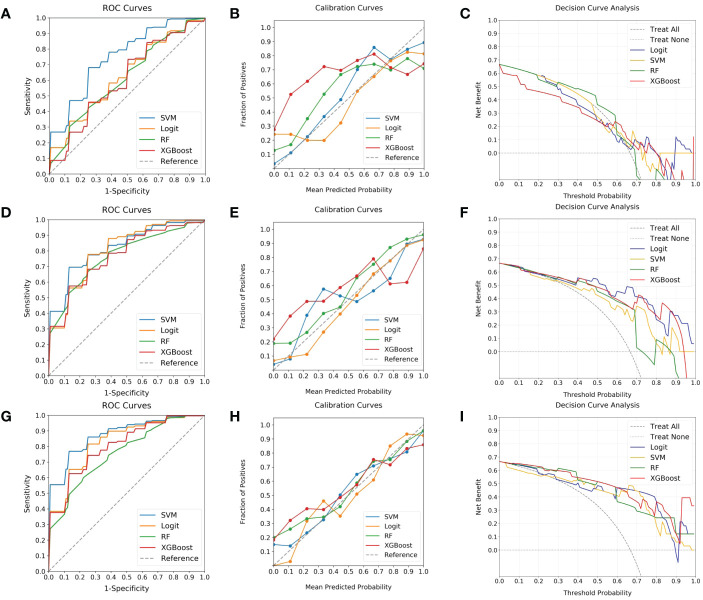
Evaluation of ML classifiers using various ROIs. **(A–C)** depict the performance characteristics (ROC, calibration, and DCA curves) of four ML classifiers (SVM, Logit, RF, and XGBoost) in the context of intratumoral ROI, achieving respective AUCs of 0.75, 0.63, 0.62, and 0.61. **(D–F)** present the same classifiers’ efficacy as applied to peritumoral ROI, with corresponding AUCs of 0.82, 0.80, 0.76, and 0.77. **(G–I)** illustrate the performance when utilizing combined ROI, where AUCs ascend to 0.88, 0.83, 0.75, and 0.81. Among the classifiers, SVM is highlighted for its superior discrimination, calibration, and comparable DCA curve, endorsing it as the predominant model.

To identify the optimal predictive model for elevated Ki-67 expression in BC patients, four ML classifiers (SVM, Logit, RF, and XGBoost) were examined. These were systematically applied to intratumoral ROI, peritumoral ROI, and their combination. The respective ROC, calibration, and DCA curves are delineated in **Figure 5**. It indicated that classifiers employing peritumoral ROI demonstrated superior discrimination ability in contrast to those utilizing intratumoral ROI (AUC: 0.76-0.82 vs. 0.61-0.75, Delong test P < 0.05). Moreover, ML models utilizing combined ROI exhibited the highest discrimination (AUC: 0.75-0.88), with Logit and SVM achieving AUCs of 0.83 and 0.88, respectively. However, the SVM model exhibited better calibration, whereas the Logit model tended to over-approximate probabilities in proximity to the 50% threshold. With comparable performance on DCA curves, the SVM classifier was thus recommended as the most efficacious model for anticipating the likelihood of heightened Ki-67 expression.

### External verification

**Figure 6:**
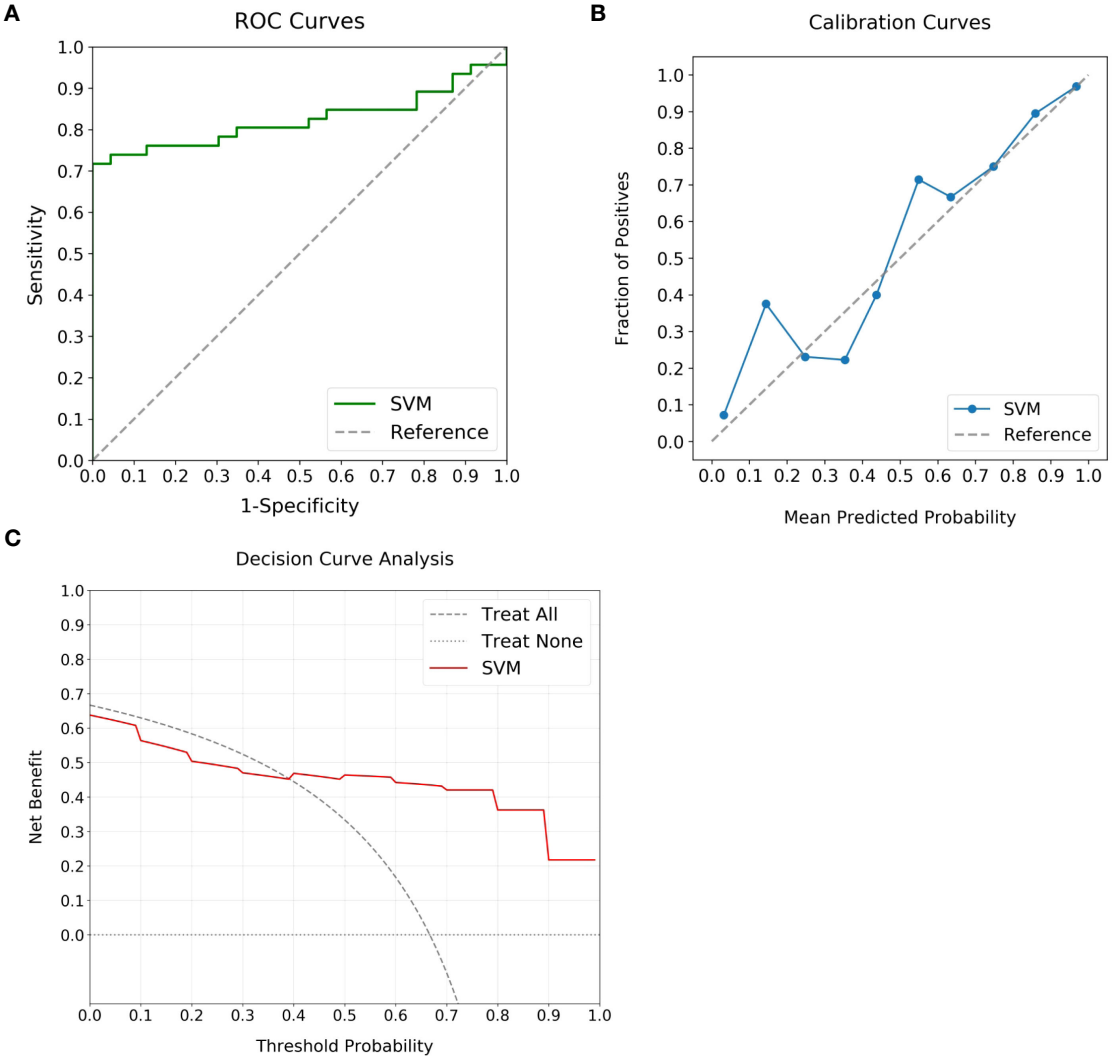
Performance analysis of the optimal ML model using external validation cohort. **(A)** displays the ROC curve, achieving an AUC of 0.82, which marks the substantial discriminatory power of the model. **(B)** highlights the calibration curve, revealing alignment between predicted likelihoods and observed events for predictions over 40%. **(C)** outlines the DCA, highlighting the clinical advantage when the threshold probability is above 40%.

In evaluating the predictive capability of the SVM model, the external validation cohort was utilized. By integrating the selected intra- and peritumoral radiomics features, the model enabled the automatic calculation of high Ki-67 likelihood for individual patients. Subsequent analysis of these computed probabilities against the actual Ki-67 expression status was performed using ROC, calibration, and DCA curves, as shown in **Figure 6**. Although exhibiting a slight reduction in performance compared to the training cohort, the SVM model continued to demonstrate significant discriminative abilities, attaining an AUC of 0.82 (**Figure 6A**). The calibration curve indicated alignment between predicted and actual occurrences when the probability was above 40% (**Figure 6B**). The DCA added confirmation of the model’s robustness, exhibiting significant net benefits when threshold probability was greater than 40% (**Figure 6C**). These findings enhanced the potential of the SVM model for high Ki-67 expression prediction.

### Model interpretation

**Figure 7:**
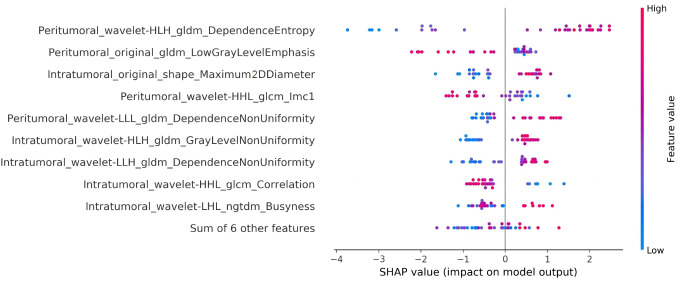
Illustration of the radiomics features associated with Ki-67 expression in the SVM model using SHAP analysis. This summary plot blends SHAP values to visually show how individual features together affect the model’s predictions. Each dot stands for a patient, with color change from blue to red indicating the feature values: red for higher and blue for lower values. The horizontal position of the dots explains the SHAP value, where a positive value suggests a higher chance of increased Ki-67 expression, while a negative value suggests the opposite. The x-axis placement of each dot reflects the impact of the respective feature on a particular patient’s prediction, thereby highlighting the correlation between higher values of the top five key features and a greater likelihood of elevated Ki-67 expression.

**Figure 8:**
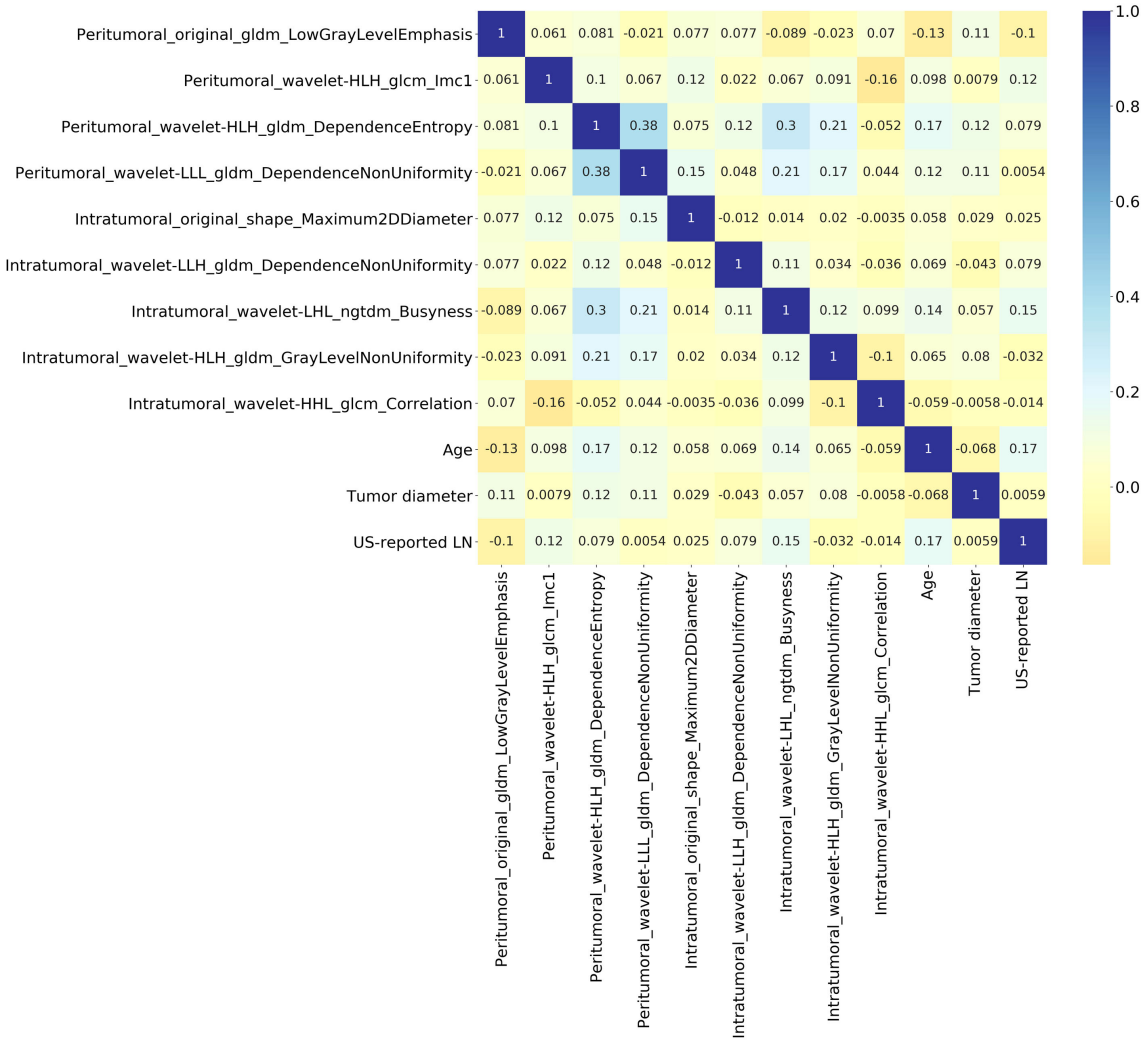
Heatmap illustrating the collinearity among the influential radiomics features in SVM model, and their associations with selected independent clinical predictors of Ki-67 expression. It reveals minimal mutual correlation, with the highest correlation coefficient not exceeding 0.4.

The interpretation of the SVM model was conducted using SHAP analysis, quantifying the individual contributions of features within the model. The calculation of absolute mean SHAP values led to the ranking of features, highlighting four radiomics features from peritumoral ROI and one from the intratumoral ROI as the five most influential determinants. A summary plot, integrating these SHAP values, was devised for visual representation (**Figure 7**), thereby providing a comprehensive insight into the role each feature assumed in predicting patient outcomes. Furthermore, to elucidate the implications of each feature, detailed descriptions of each feature in SHAP analysis are provided in **Supplementary Table S1**. Concurrently, the collinearity among these influential radiomics features, and their associations with selected independent clinical predictors, was analyzed and illustrated in a heatmap, as presented in **Figure 8**. This analysis revealed minimal mutual correlation, with the highest correlation coefficient not exceeding 0.4, indicating a low degree of collinearity among the selected radiomics features and clinical predictors.

## Discussion

The precise and dynamic determination of Ki-67 status is crucial for optimizing treatment strategies in BC patients. Elevated Ki-67 expression often correlates with adverse prognosis but may enhance responsiveness to chemotherapy (27, 28). While many studies have used radiomics to predict Ki-67 levels, they have largely focused on the tumor extent, overlooking essential information in the immediate peritumoral environment (29–31). Moreover, existing research, primarily focused on MRI of intra- and peritumoral regions, has failed to incorporate US-based radiomics, thereby limiting the wider applicability and repeatability of these studies (32–34). The current study addressed this gap by developing an ML prediction model using US radiomics features. An SVM model utilizing combined intra- and peritumoral radiomics features finally proved superior for predicting Ki-67 levels. This methodology may pave the way for a widely applicable, repeatable, and non-invasive evaluation process in personalized BC diagnosis and treatment.

To the best of our understanding, this study marked a first in employing ML models to predict Ki-67 levels using US-based intra- and peritumoral radiomics features. The incorporation of the S-Detect auxiliary diagnostic system, based on a deep convolutional neural network, ensured accurate differentiation of tumor boundaries without manual human input, and overcame challenges such as ultrasonic artifacts and speckles, thereby enhancing the precision and efficiency in clinical applications of radiomics (35, 36). Through careful evaluation and comparison, the optimal ML model selected in this study may mitigate the necessity for frequent CNBs for Ki-67 assessment, serving as a valuable **Supplemental Tool**. Importantly, it acknowledges the variations in Ki-67 expression across different tumor areas in BC, and thus is not affected by significant cellular proliferation heterogeneity (37). When integrated with core needle tissue sampling, this approach may provide clinicians with a more precise instrument for individualized decision-making, underscoring the potential of this methodology in clinical practice.

Utilizing the SHAP interpretation methodology, the critical elements of our chosen SVM model were identified, comprising four peritumoral and one intratumoral radiomics feature. Briefly, the SHAP methodology allocates a value to each feature, signifying the influence of that feature on the model’s prediction relative to a baseline, thus enhancing model interpretability (38). The insights obtained from the SHAP analysis revealed a robust association between elevated Ki-67 levels and the heterogeneity surrounding the tumor, a finding in line with earlier research (33, 39). The prominence of peritumoral features in our model supports the notion that regions adjacent to the tumor may offer enhanced predictive insight into Ki-67 expression (33, 39). Specifically, these peritumoral regions often exhibit complex cellular interactions and microenvironment changes that may reflect the aggressiveness of the tumor, thereby serving as significant indicators for predicting Ki-67 levels (40). This suggests that peritumoral features are not merely supplementary but hold intrinsic predictive value, offering a broader perspective on Ki-67 expression. Together with intratumoral features, they form a complementary framework that may lead to more accurate and individualized predictions (41, 42). However, it is notable that although we can identify some radiomics features with statistical relevance to clinical outcomes from a variety of categories, many of these belong to texture and higher-order statistical features. These features are fundamentally abstract, being mathematical descriptions derived from imaging data. The data-driven nature of radiomics may not directly reflect the underlying biological processes, making the elucidation of the biological mechanisms linking these features to clinical outcomes challenging at present (43).

Our study confirmed correlations between elevated Ki-67 levels in BC cases and associated clinical factors such as advanced age, larger lesion size, and susceptibility to axillary lymph node metastases, aligning with previous findings (18, 44, 45). Despite these insights, the predictive accuracy of these clinical features remained limited, evidenced by an AUC of merely 0.709. This was notably inferior to the AUC of 0.82 achieved through US-based radiomics features, highlighting the challenge of relying solely on traditional clinical parameters for precise Ki-67 level prediction. In studies focusing on intra- and peritumoral radiomics features, Li et al. (32) and Jiang et al. (33) demonstrated predictive accuracies of 0.749 and 0.838 based on MRI images, respectively. This underlines that our model, centered on intra- and peritumoral US radiomics features, also possesses strong predictive power, warranting further clinical exploration.

Despite the promising findings, our study acknowledges several limitations that require attention. The single-center, retrospective design, along with a restricted patient cohort, could inhibit the wider application of our conclusions. Additionally, inconsistencies in US settings among different institutions might negatively affect the performance of the models. This challenge is further compounded by the decision to restrict the inclusion criteria to lesions with a singular visible mass on US, precluding the extension of our findings to non-mass and multi-focal lesions. Another limitation lay in the lack of evaluation of other prevalent US modalities, such as elastography or contrast-enhanced US, representing an additional limitation and a field for future investigation. A significant aspect of our methodology that called for further investigation was our choice of a 3 mm radial extension from the tumor margin to expand the initial ROI. This decision aimed at balancing the optimal accuracy of a 2-4mm peritumoral region size as recommended by Ding et al. (24), while minimizing the occurrence of segments extending beyond the skin. Future studies should probe into the predictive value of peritumoral regions with varying dilation distances in relation to Ki-67 levels to better understand the implications of this parameter. Lastly, our ML model lacked external validation from additional centers, indicating a necessity for further validation. Despite these barriers, the study does highlight the potential utility of radiomics-based ML models in predicting Ki-67 levels of BC patients. This insight emphasizes the need for future research, specifically through multi-center, prospective studies to enhance the reliability and practicality of the model.

## Conclusion

The present study highlighted the capability of ML models, notably the SVM model utilizing radiomics features from both intra- and peritumoral US images, to predict elevated Ki-67 levels in BC patients. The model demonstrated consistent and reliable performance in both internal and external verifications, indicating its promise as a noninvasive preoperative prediction method. Serving as a valuable supplement to CNB, this approach is anticipated to guide treatment strategies and contribute to personalized clinical decision-making for BC patients.

## Data availability statement

The raw data supporting the conclusions of this article will be made available by the authors, without undue reservation.

## Ethics statement

The studies involving humans were approved by the Institutional Review Board of Jiading District Central Hospital Affiliated Shanghai University of Medicine & Health Sciences (2023K29). The studies were conducted in accordance with the local legislation and institutional requirements. Written informed consent for participation was not required from the participants or the participants' legal guardians/next of kin because of the retrospective nature of this research.

## Author contributions

JW: Conceptualization, Data curation, Formal Analysis, Investigation, Methodology, Validation, Visualization, Writing – original draft. WG: Formal Analysis, Investigation, Validation, Writing – original draft. ML: Formal Analysis, Investigation, Software, Supervision, Writing – review & editing. XY: Formal Analysis, Investigation, Methodology, Writing – review & editing, Funding acquisition. DY: Conceptualization, Data curation, Formal Analysis, Funding acquisition, Investigation, Project administration, Resources, Writing – original draft, Writing – review & editing.
