# Supplementary material for: Development of an interpretable machine learning model for Ki-67 prediction in breast cancer using intratumoral and peritumoral ultrasound radiomics features
Source: Front Oncol. 2023 Nov 17;13:1290313. doi: 10.3389/fonc.2023.1290313 (PMC10691503; doi:10.3389/fonc.2023.1290313)
Supplement: Supplementary file 1 [file Table_1.docx]

**Supplementary**

**Table S1**. Detailed descriptions of radiomics features utilized in SVM model for Ki-67 estimation using SHAP analysis

| Position | Filter | Type | Name | Description |
| --- | --- | --- | --- | --- |
| Peritumoral | wavelet-HLH | gldm | DependenceEntropy | Measures the randomness/variability in the co-occurrence of dependent pixels within an image. |
| Peritumoral | original | gldm | LowGrayLevelEmphasis | Emphasizes regions with lower gray-level values, indicating the presence of darker textures. |
| Intratumoral | original | shape | Maximum2DDiameter | Represents the largest 2D diameter length within the region of interest. |
| Peritumoral | wavelet-HHL | glcm | Imc1 | A measure of the informational correlation among image texture. |
| Peritumoral | wavelet-LLL | gldm | DependenceNonUniformity | Reflects the similarity of dependencies in the image, where lower values indicate more uniform dependencies. |
| Intratumoral | wavelet-HLH | gldm | GrayLevelNonUniformity | Measures the variation of gray-level intensity values in the image, indicating the heterogeneity in intensity. |
| Intratumoral | wavelet-LLH | gldm | DependenceNonUniformity | Reflects the similarity of dependencies in the image, where lower values indicate more uniform dependencies. |
| Intratumoral | wavelet-HHL | glcm | Correlation | Indicates the linear dependency of gray-level values in the image. |
| Intratumoral | wavelet-LHL | ngtdm | Busyness | Reflects the local intensity variation, where higher values indicate more complex texture patterns. |

glcm: Gray-Level Co-Occurrence Matrix, gldm: Gray-Level Dependence Matrix, ngtdm: Neighbouring Grey Tone Difference Matrix; In the wavelet-transformed features, the low and high-frequency coefficient blocks are denoted as L and H, respectively.
